# The Role of Immune Checkpoint Molecules on Macrophages in Cancer, Infection, and Autoimmune Pathologies

**DOI:** 10.3389/fimmu.2022.837645

**Published:** 2022-03-28

**Authors:** Victoria C. Brom, Christof Burger, Dieter C. Wirtz, Frank A. Schildberg

**Affiliations:** Clinic for Orthopedics and Trauma Surgery, University Hospital Bonn, Bonn, Germany

**Keywords:** macrophage, myeloid cell, checkpoint inhibitor, cancer, infection, autoimmune disease, immunotherapy, immunodiagnostics

## Abstract

Immune checkpoint inhibitors have revolutionized immunotherapy against various cancers over the last decade. The use of checkpoint inhibitors results in remarkable re-activation of patients’ immune system, but is also associated with significant adverse events. In this review, we emphasize the importance of cell-type specificity in the context of immune checkpoint-based interventions and particularly focus on the relevance of macrophages. Immune checkpoint blockade alters the dynamic macrophage phenotypes and thereby substantially manipulates therapeutical outcome. Considering the macrophage-specific immune checkpoint biology, it seems feasible to ameliorate the situation of patients with severe side effects and even increase the probability of survival for non-responders to checkpoint inhibition. Apart from malignancies, investigating immune checkpoint molecules on macrophages has stimulated their fundamental characterization and use in other diseases as well, such as acute and chronic infections and autoimmune pathologies. Although the macrophage-specific effect of checkpoint molecules has been less studied so far, the current literature shows that a macrophage-centered blockade of immune checkpoints as well as a stimulation of their expression represents promising therapeutic avenues. Ultimately, the therapeutic potential of a macrophage-focused checkpoint therapy might be maximized by diagnostically assessing individual checkpoint expression levels on macrophages, thereby personalizing an effective treatment approach for each patient having cancer, infection, or autoimmune diseases.

## Introduction

The importance of the field of immunotherapy is well recognized in treating various diseases. Recently, the success of immune checkpoint inhibition has revolutionized therapeutic options, especially in oncology. Blockade of the so-called checkpoint molecules is crucial in regulating immune reactions, as these co-receptors modulate immune responses following antigen presentation. Ideally, this form of therapy provides effective immune reaction on the one hand and allows maintenance of sufficient self-tolerance while preventing autoimmunity and avoiding excessive immune cell activation on the other.

In general, immune reaction to pathogens results from major histocompatibility complex (MHC)-bound antigen presentation specifically activating T cells, which then regulate the quality and duration of immune responses. At the same time, secondary molecules make a further impact on the interaction between antigen-presenting cells (APCs) and T cells; for example, immune checkpoint molecules on the surface of T cells balance this pathway as co-stimulatory or co-inhibitory receptors (1, 2).

Innovative checkpoint inhibitors do not aim to directly kill pathogenic cells as is the purpose of chemo- or radiotherapy, instead focus on regaining immune competence to restore endogenous anti-tumor activity (3, 4). These monoclonal antibodies function by suppression of immune checkpoints that act as co-regulatory receptors and limit immune responses, which in turn leads to thorough activation of the immune system (5, 6). Complex dynamic immune responses following checkpoint inhibition are particularly accomplished by downregulating the activation of naïve T cells, resulting in enhanced immunity and maintenance of self-tolerance (3). Therefore, this therapeutic modulation can be visualized as a gas and brake pedal for immune system regulation, because the possible outcomes are enhanced or restrained immune responses as a consequence of either promoting or restricting stimulatory and inhibitory pathways.

So far, checkpoint inhibition is used in cancer therapy to control T cell function and has proven to be effective against a broad spectrum of cancer types (7). Immunotherapy offers a highly promising anti-cancer strategy that has already achieved remarkable clinical benefit in, e.g., malignant melanoma, non-small cell lung cancer, renal and bladder cancers (8); hence, the use of immune checkpoint inhibitors across the spectrum of human cancers is rapidly expanding. Despite the reasonable success of checkpoint therapy, many patients are non-responders to checkpoint therapy, and only a minority achieve sufficient anti-cancer response (9, 10). Currently, the unlikeliness of immunologically cold tumors responding to immune checkpoint blockade is attributed to upregulation of alternative immune checkpoint pathways on T cells (11).

For this reason, there is an urgent need to improve checkpoint immunotherapy. The limitations could already be partly overcome by combination of different checkpoints for immune checkpoint blockade, e.g., CTLA-4 and PD-L1 to restore T cell effector function (12). Still, only few patients respond and experience sufficient anti-tumor immunity despite increased cytotoxicity in combination therapy. Moreover, this leads to more severe side effects, such as inflammatory reactions in the brain, gastrointestinal organs and cardiovascular system (13–15). Consequently, the current knowledge has to be further expanded. At present, certain checkpoints are widely inhibited on all their expressing cells. In contrast to this broad approach, it seems highly advantageous to perform immune checkpoint inhibition (ICI) cell-type specifically. When focused on one immune cell that is relevant for disease progression and simultaneously expressing the targeted checkpoint molecule, such an individualized treatment may promote remarkable immunity. At the same time, it might reduce adverse events originated from manipulation of the entire immune system. Even stimulation of checkpoint proteins on specific immune cells may be a promising therapeutic option in the future, as for some diseases it is not overexpression but rather a lack of checkpoint regulation causing illness.

In this review, macrophages will be discussed as another cell population contributing to immune reaction, given their expression of co-stimulatory and co-inhibitory checkpoint molecules. Unfortunately, even today, it is difficult to accumulate data regarding immune checkpoint targeting on mononuclear phagocytic cells, because evidence mostly comes from coincidental findings. However, as some pieces of information can be combined, new exciting application areas emerge and create further possibilities for beneficial therapy *via* checkpoint modulation.

Therefore, we discuss in detail how cancer immunotherapy can be improved by considering checkpoint inhibition on macrophages. This may potentially allow to enable advanced personalized treatment and use levels of checkpoint expression as predictive biomarkers. Treatment outcomes are already being anticipated by examination of checkpoint expression on T cells, potentially being transferable to macrophages as well. Beyond that, we speculate whether focusing checkpoint regulation through ICI or the use of novel agonistic antibodies on cell types other than T lymphocytes may hold potential to treat pathologies aside from malignancy, e.g., infectious diseases in acute or chronic phases.

## Co-Inhibitory and Co-Stimulatory Molecules on Macrophages in Cancer

With the focus on malignancies, the organization of an immunosuppressive microenvironment to undercut the immune system is considered a hallmark of cancer (4). Besides T cells, cells of the myelomonocytic lineage are also known to be contributing factors to this immunosuppressive environment and are therefore essential targets for cancer cells that evade immune clearance.

**Figure 1:**
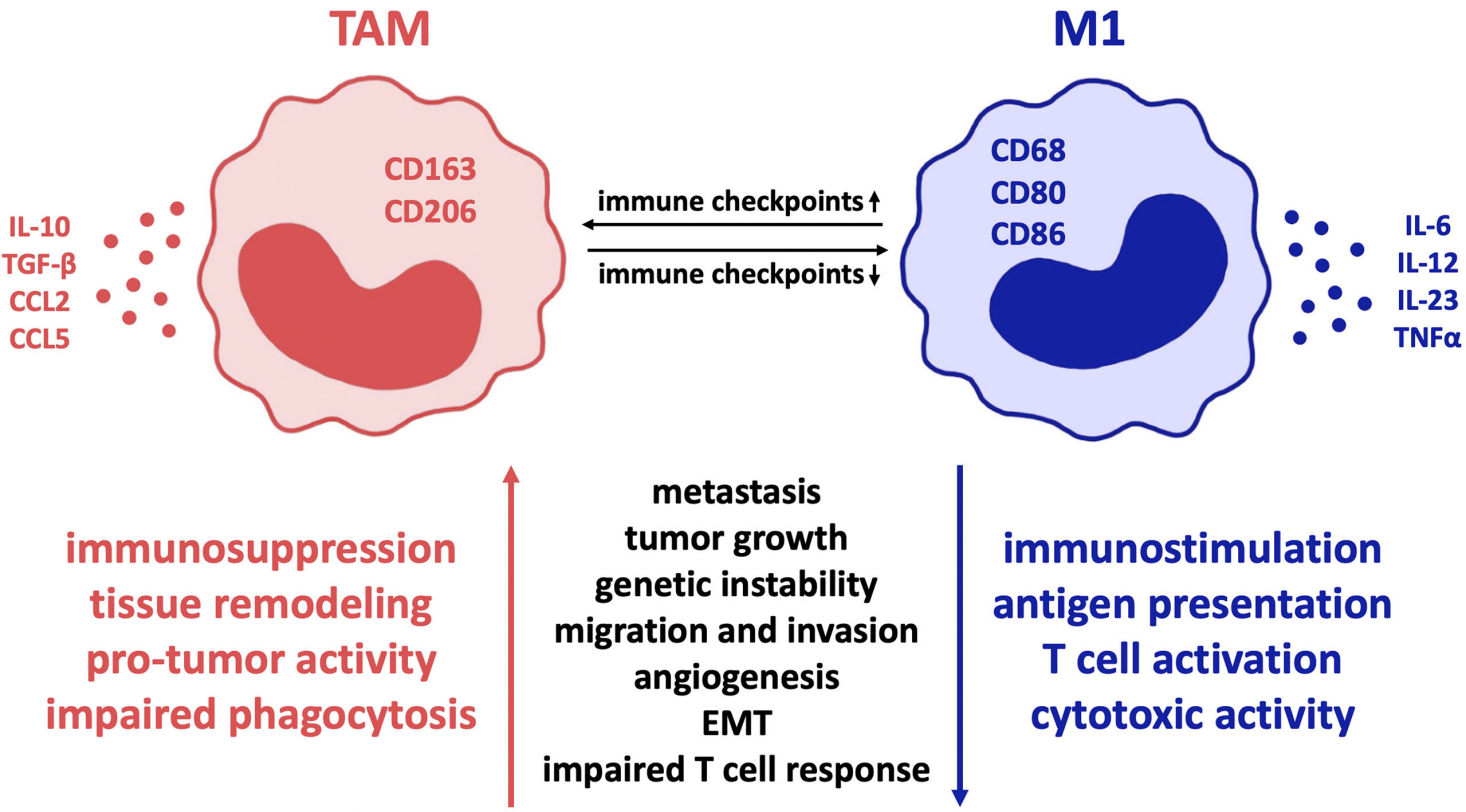
Influence of co-regulatory immune checkpoint molecules on macrophage polarization in cancer. In various malignant diseases, the expression of such co-receptors is proven to alter tumor-associated macrophages towards the so-called M2 immune profile with reduced inflammation and thereby mostly “pro-tumor” activity. In contrast, minor expression of immune checkpoints correlates with the M1 macrophage type, characterized by cytotoxic immune cell activity and improved phagocytic ability that results in significant disease clearance. Therefore, inhibition of immune checkpoint expression on macrophages is a highly promising treatment strategy in cancer pathologies. Regarding colorectal cancer, pancreatic cancer and glioblastoma, predominantly PD-1 and CD47 are of great relevance and offer promising targets for checkpoint inhibition. Though, due to the fact that macrophages in different diseases are characterized by expression of different immune checkpoints, the importance of individual therapeutic approaches is highlighted. TAM, tumor-associated macrophages; EMT, epithelial mesenchymal transition.

The expression of a variety of co-regulatory receptors on macrophages, such as PD-L1, PD-L2, CTLA-4 ligands B7-1 and B7-2, Tim-3, CD47, V-domain Ig suppressor of T cell activation (VISTA), and B7-H4 (16–18), has been shown to correlate with exhausted T cell phenotypes and therefore with an immunosuppressive tumor environment and poor clinical outcome (18–20). Given that checkpoint receptors are broadly upregulated in tumor-associated macrophages (TAM) (21), which contribute to the main aspects of malignancy such as immune suppression, metastasis, invasiveness, angiogenesis, and therapeutic resistance (5, 22, 23), it seems quite likely that these findings depict causality of checkpoint expression on macrophages and macrophage-derived tumor promotion. As a consequence, it is to be assumed that macrophages can be regulated by checkpoint inhibition. This serves the intention of reversing TAM-provided tumor promotion and enhances macrophages’ anti-tumor immune potential (17). Thus far, there is evidence that checkpoint inhibitors cause a change in macrophage polarity from M2- to M1-phenotype (**Figure 1**). This results in sufficient immunity against cancer cells (17, 19, 23), as M1 macrophages are pro-inflammatory in contrast to the mostly anti-inflammatory, hence pro-tumor M2 macrophage phenotype (18, 22, 24).

TAM originate from newly recruited monocytes as well as resident tissue-specific macrophages through microenvironmental stimuli such as chemokines, cytokines, extracellular matrix components and hypoxia (17, 25). Due to their origin from independent specific lineages and therefore, great plasticity, TAM are highly heterogenous and subsequently exceedingly difficult to differentiate (26). Characterization of TAM is further complicated as a result of constant adaptation to environmental stimuli, lack of specific markers in between populations and differences between human and animal experiments as well as between *in vitro* and *in vivo* studies (27–29).

In general, however, M1 TAMs are defined by expression of CD68, CD80 and CD86 and secretion of pro-inflammatory cytokines such as tumor necrosis factor α (TNFα), interleukin (IL)-1α, IL-1β, IL-6, IL-12, IL-18, and IL-23 as well as nitric oxide (NO) synthase that contribute to eliminating tumor cells (30–32). On the contrary, M2 TAMs express CD163 and CD206 (27, 33) and secrete IL-10, TGF-β, CCL2, CCL5 (30, 31) and IL-13 that maintain the immune-suppressive environment (5). It is further shown that in contrast to M1 macrophages, TAM usually express high levels of checkpoint proteins in most cancer diseases (34). Though, depending on the specific disease, macrophage subtypes vary significantly and co-expression of additional molecules is often noticed (22). The shift in macrophage polarity towards the inflammatory M1 type can be induced by cytokines associated with inflammation and removal of tumor cells and pathogens, e.g., IFN-γ, LPS, TNFα, and GM-CSF (17). Moreover, checkpoint molecules can stimulate polarization towards the inflammatory M1 type by secreting proinflammatory cytokines such as IL-1, IL-12, TNFα, MIP1a, and NO as Hoves et al. showed macrophages being modulated towards the pro-tumor phenotype due to application of CD40 (35). In many cases, it is confirmed that transformation of macrophages from M2- to M1-phenotype is sufficient to cause an anti-tumor immune response (22, 36, 37).

Based on the following examples of aggressive cancer pathologies with overall high mortality and need for improvement of the currently insufficient treatment options, we have attempted to explain the influences of co-regulatory receptors on macrophage function, with particular focus on their phagocytic ability. Thereby, certain recurring patterns become apparent, allowing predictions about the way other checkpoint molecules could affect anti-tumor immunity and how this may be used to improve cancer therapy in the future.

### Colorectal Cancer

Colorectal cancer is an important disease in this regard, as patients rarely show clinical symptoms in early stages when standard treatment options could provide satisfactory effects. Survival rates drastically decrease in advanced stages of cancer as metastasized cancer is often incurable (26, 38, 39).

There is evidence for expression of checkpoint molecules on TAMs (**Figure 1**). Gordon et al. provided new insights into PD-1 expression by restricting innate and adaptive immune reactions in human colorectal cancer and mouse colon cancer (**Table 1**). In both mouse and human TAMs, high expression levels of PD-1 were detected that correlated with reduced phagocytosis and therefore with disease severity (40). Such findings in murine models can be explained by contrary TAM polarization states: The majority of PD-1^+^ TAMs show the pro-tumor M2-macrophage profile, whereas PD-1-deficient TAMs express the inflammatory, anti-tumor M1-immunoprofile (40). However, PD-1 deficiency leads to significantly lower tumor burden because of increased phagocytic ability, which implies that regular TAM function can be re-established. PD-1 was previously known to restrain various immune cells in the tumor microenvironment (including T cells, B cells, NK cells, and DCs), but it can now also be applied to macrophages based on recent information.

**Table 1:** Summary of the effects that up- and downregulation of immune checkpoint expression have on macrophage polarity and resulting consequences in malignant, infectious, and autoimmune diseases following up- or downregulation of immune checkpoint expression.

	CHECKPOINT MOLECULE	EXPRESSION ↑ / STIMULATION	EXPRESSION ↓ / INHIBITION	POSSIBLE THERAPY	REFERENCES
**CANCER**					
colorectal cancer	PD-1	disease progression (M2)	TAM phagocytosis (M1)	ICI	(25)
	CD47	disease progression (M2)	TAM phagocytosis (M1)	ICI	(26–28)
pancreatic cancer	PD-1	disease progression (M2)	TAM phagocytosis (M1)	ICI	(17, 29–32, 34)
	VISTA	disease progression (M2)	TAM phagocytosis (M1)	ICI	(29, 31–33)
	CD47	disease progression (M2)	TAM phagocytosis (M1)	ICI	(34)
glioblastoma	CD47	disease progression (M2)	TAM phagocytosis (M1)	ICI	(12, 13, 38–40)
	PD-1	disease progression (M2)	TAM phagocytosis (M1)	ICI	(37, 41–43)
	CD73	disease progression (M2)	TAM phagocytosis (M1)	ICI	(41, 44–47)
	CTLA-4	disease progression (M2)	TAM phagocytosis (M1)	ICI	(41–43)
**INFECTION**					
**chronic viral**					
HBV/HCV	PD-1	viral persistence (M2)	inflammatory immune reaction (M1)	ICI	(2, 48–51)
	Tim-3	viral persistence (M2)	inflammatory immune reaction (M1)	ICI	(2, 48, 50–54)
HIV	PD-1	viral persistence (M2)	inflammatory immune reaction (M1)	ICI	(55–58)
	Tim-3	inflammatory immune reaction (M1)	HIV production (M2)	stimulation	(59)
	VISTA	viral persistence (M2)	inflammatory immune reaction (M1)	ICI	(60)
**acute viral**					
Influenza A	Tim-3	viral persistence (M2)	inflammatory immune reaction (M1)	ICI	(61–63)
**bacterial**					
tuberculosis	PD-1	dual role	dual role		(64–68)
	Tim-3	disease progression (M2)	inflammatory immune reaction (M1)	ICI	(69)
sepsis	PD-1	disease progression (M2)	inflammatory immune reaction (M1)	ICI	(67, 70–76)
**AUTOIMMUNITY**					
MS/EAE	VISTA	phagocytosis, improved survival	disease progression	stimulation	(60, 77–83)
	PD-1	improved survival	disease progression	stimulation	(49, 84, 85)
	CD47	reduced phagocytosis, disease progression	phagocytosis, inflammatory immune reaction	ICI	(86)
atherosclerosis	CD47	disease progression (M2)	inflammatory immune reaction (M1)	ICI	(87–89)
	PD-1	tbd	disease progression (M2)	stimulation	(90–93)
	Tim-3,-4	tbd	disease progression	stimulation	(93–95)
	GITR	disease progression (phagocytosis)	improved survival	ICI	(96–100)
diabetes type 1	CD47	improved survival (limited phagocytosis, M2)	disease progression (phagocytosis)	stimulation	(101–104)
	PD-1	improved survival (limited phagocytosis, M2)	disease progression	stimulation	(105)

As checkpoint expression has specific consequences in a disease, individual treatment options can be deduced. Depending on the characteristics of the pathology and each patient’s expression pattern, either promotion of a particular immune checkpoint expression or restriction via immune checkpoint inhibition (ICI) displays potential therapeutical options. In addition, even combined stimulation or inhibition of certain expression rates can be applied to improve disease severity and therefore ameliorate patients’ wellbeing. ICI, Immune checkpoint inhibition; HBV, Hepatitis B virus; HCV, Hepatitis C virus; HIV, Human immunodeficiency virus; MS, Multiple sclerosis; EAE, Experimental autoimmune encephalomyelitis; tbd, to be defined.

Besides PD-1, the CD47-SIRPα-axis seems to be a promising target in colorectal cancer. In addition to enhancing T cell activation to improve anti-cancer immunity (41), the checkpoint molecule CD47 acts as an efficient “don’t eat me” signal. Moreover, its binding to SIRPα on macrophages and dendritic cells (DC) results in drastically reduced phagocytosis, allowing tumor progression (M2) (44). Tumor cells take advantage of this potent mechanism to evade immune clearance and thereby avoid being destroyed by phagocytic cells. Similar to what is seen in modulating PD-1 in colorectal cancer, inhibiting the CD47 checkpoint pathway has been shown to restore function of macrophages and thus led to growth inhibition and regression of tumor cells in experimental models (44, 45). Presumably, improved phagocytosis following CD47 inhibition is due to a change in macrophage polarization towards the inflammatory M1 phenotype.

Overall, immune checkpoint inhibition represents an innovative therapeutic approach for colorectal cancer, as antibodies inhibiting PD-1 and CD47 will restore patients’ immune competence and regain the ability to eliminate cancer cells. Combination of different immune checkpoint inhibitors may be an option to treat more severe cases of colorectal cancer, though will most likely be accompanied by extensive adverse effects.

### Pancreatic Cancer

Patients with pancreatic cancer develop no or merely unspecific symptoms until the disease has considerably advanced, and simultaneously show early metastatic spread. Current surgical and radiation therapies are only feasible in non-metastatic forms of pancreatic cancer. Because of these limited therapeutic options, the survival rates can be as low as 5% (21, 42, 43, 46, 47, 106, 107).

About 12.5% of pancreatic cancer patients express PD-L1, which, when binding to PD-1, leads to T cell anergy and apoptosis, resulting in cancer cells being able to evade the immune system (106, 108). Until today, solely targeting the PD-1-PD-L1 axis has however not been successful in pancreatic cancer (21, 46). Despite that, significant reduction of tumor progression can be achieved by combined administration of inhibitory antibodies targeting PD-1 and BAG-3 (109).

The expression of VISTA as another checkpoint molecule has been reported on CD68^+^ macrophages in pancreatic ductal adenocarcinoma (PDAC) at an even higher rate than the structurally similar PD-1 (46, 48). VISTA^+^ cells showed an anti-inflammatory M2 macrophage phenotype and therefore present a pro-tumor profile. VISTA on macrophages is likely to be a promising target for immune checkpoint inhibition, because it is expressed most often in human PDAC (48) and reversing the M2 phenotype to an M1-like profile through VISTA ICI will result in an inflammatory anti-tumor reaction.

The different expression patterns on macrophages may in part explain why anti-PD-1 antibodies alone do not result in sufficient anti-cancer immunity. VISTA and PD-1 as checkpoints represent separate inhibitory pathways. Despite anti-PD-1 therapy enhancing T cell function, this alone is considered not powerful enough in a pro-tumor microenvironment and would require the immune status to be changed beforehand. Supporting the hypothesis of rather complex checkpoint regulation in particularly immune-evasive PDAC, VISTA is co-localized with PD-1/PD-L1 and other immune checkpoints in some cases of pancreatic cancer (46), correlating with impaired immune cell function.

Their synergistic role in immune evasion of pancreatic cancer has been verified in a murine model as a combined anti-VISTA and anti-PD-L1 application resulted in improved conditions (110). As a result of this co-targeting and its significant association with better disease management and longer survival, combination therapy could be an option to maximize profit of their individual effects. Moreover, expression rates may be used to predict therapeutic outcome.

Hou et al. shared the concept of macrophage-expressed VISTA playing an important role in immune evasion of pancreatic cancer: VISTA expression is predominantly higher on CD68^+^ macrophages than on CD3^+^ T cells and CD19^+^ B cells. Recently, anti-VISTA antibody has been shown to significantly reduce the number of liver metastases in PDAC, explained by improved phagocytic ability of the macrophages. The anti-tumor immunity following anti-VISTA treatment is suspected to be a consequence of altered macrophage polarization towards the inflammatory M1 phenotype (43), as VISTA’s biological function in pancreatic cancer is still unclear (106).

Beyond that, targeting the checkpoint molecule CD47 led to similar results in a mouse model of PDAC, as CD47 on cancer cells inhibits macrophage phagocytosis, causing M2 macrophage polarization (111). Checkpoint blockade provided remarkable immune reaction against pancreatic tumor cells, in particular when synergistically targeting CD47 and PD-L1.

Comparing the influence of checkpoint molecules on macrophages in colorectal and pancreatic cancer is intricate as a consequence of macrophage heterogeneity between diseases and moreover, lack of extensive systematic analyses. Despite evidence of similar effects for PD-1 and CD47 causing pro-tumor macrophage polarization, data regarding VISTA on colorectal cancer is not yet available.

### Glioblastoma

Glioblastoma is characterized by severe brain edema, necrosis, as well as midline shift. Mid-survival rates are about 1 year under treatment, since surgical resection is impossible and radio-chemotherapy provides insufficient results. Unfortunately, checkpoint inhibition as glioblastoma treatment has been rather unsuccessful and does not show significant advantages over existing therapeutic options (37, 112).

Nevertheless, findings of a recent study (37) indicate that immune checkpoints do play a significant role in the pathology of glioblastoma and might be a promising therapeutic approach if targeted specifically. In cases of glioblastoma multiforme (GBM), a synergistic cancer killing effect of rapamycin and hydroxychloroquine (RQ) was reported. This is explained by RQ causing TAM reprogramming from M2-equivalent pro-tumor to inflammatory M1 polarization. Microglial cells function as macrophages of the brain and similarly present either a neuroprotective or neurotoxic phenotype in the tumor microenvironment, depending on environmental stimuli. Transitioning from minimal immune-activated TAM phenotype leads to enhanced phagocytosis levels in tumor-associated microglia (49), which results in increased immune responses and improved checkpoint blockade targeting PD-1/PD-L1 (52). In addition, RQ combined with PD-1-checkpoint inhibition enhances the intra-tumoral M1/M2 ration, CD8/CD4 ratio, and the phagocytic ability.

The observed change in macrophage type can further be explained by RQ lowering the expression of CD47 and its ligand SIRPα on malignant cells and macrophages. Zhang et al. showed that inhibiting the CD47-SIRPα pathway results in improved anti-cancer immune responses in GBM as CD47 and SIRPα interaction typically restrict phagocytic ability of macrophages (M1) (53). This restriction of microglial phagocytosis is due to CD47 executing a “don’t eat me” signal in its interaction with SIRPα on malignant cells (16). Other research groups (17, 53, 54) further report that inhibiting CD47 on murine macrophages in glioblastoma results in an elevated M1:M2 ratio and thereby an inflammatory immune reaction against tumor cells. Given this information, CD47 could be used to predict checkpoint therapy outcome. In another recent glioma mouse model, CD47-checkpoint inhibition provided effective anti-tumor immunity in five aggressive pediatric brain cancers by enhancement of CD8^+^ T cell priming (50). This results from improved antigen presentation, which is probably in part caused by enhanced M1 microglia levels.

Goswami et al. examined CD73 as a checkpoint molecule often co-expressed on CD68^+^ macrophages in GBM and found CD73^+^ macrophages to be resistant to PD-1 checkpoint inhibition in glioblastoma (51). It is further shown that they promote tumor expansion through maintaining an immunosuppressive environment in GBM (55, 113). Upon investigating the immune profile in glioblastoma in either CD73^+^ or CD73-deficient mice, it became apparent that significantly more inducible nitric oxide synthase-positive (iNOS^+^) immunostimulatory macrophages than CD206^+^ immunosuppressive macrophages could be found in CD73-deficient mice. These findings of co-expressed molecules on CD73^+^ macrophages, that indicate functional characteristics but do not represent other macrophage subtypes, are contrary to what is detected in wildtype mice. It can therefore be assumed that CD73 contributes to mediating the shift in macrophage phenotypes in glioblastoma towards a pro-tumor environment (M2).

Investigation of checkpoint inhibition of PD-1 and CTLA-4 on their own as well as in combination in wildtype and CD73-deficient mice confirmed the hypothesized role of CD73 in GBM: Therapeutical outcome was superior when both checkpoints were targeted in combination as opposed to single administration, which seems plausible as they are often co-expressed in glioblastoma. Particularly improved immune response could be detected when checkpoint blockade was applied to CD73-deficient mice that are already associated with macrophages of anti-tumor immune phenotype (51).

PD-1 blockade itself only affected tumor infiltrating lymphocytes at a low level and had basically no effect on the mostly CD73^+^ myeloid cells in the tumor microenvironment of glioblastoma. Nevertheless, immune blockade of CD73 aiming to alter macrophage polarization in combination with inhibition of CTLA-4 and PD-1 to enhance T cell infiltration will favorably change the results of checkpoint therapy and most likely provide successful outcomes for GBM patients. In fact, an anti-CD73 antibody has already been proven to be beneficial in preclinical and early clinical studies (56, 57).

It is yet to be remembered that immune regulation in glioblastoma is presumably more complicated than what can be assumed at the current level of knowledge. Hence, more information is constantly gained and needs to be considered in analysis of GBM immunity. As an example, IL-12 is shown to improve immune reaction in a murine glioblastoma model (58). Triple therapy targeting IL-12, CTLA-4, and PD-1 ultimately even led to full eradication of glioma in mice because of induced M1 polarization in TAMs (59, 60). Anyhow, as the dominating immune cells at 30–50% in GBM, macrophages represent promising targets for anti-cancer immunotherapy such as checkpoint inhibition (59). Undeniably, it is essential to expand the current knowledge about expression and co-expression of relevant immunomodulatory molecules on macrophages in GBM to allow establishment of sufficient therapeutical approaches.

Finally, it can be concluded that targeting immunoregulatory molecules on defined cells, whose influence on patients´ cancer is known, and where necessary in specific combinations, may serve as a more controlled approach to checkpoint inhibition. It therefore seems possible to achieve precise anti-tumor immunity while reducing the frequency and intensity of adverse autoimmune reactions.

## Immune Checkpoint Molecules in Infectious Diseases

Co-regulatory molecules on macrophages not only play a major role in cancer but also contribute to the development and progression of infectious diseases. Therefore, we have reviewed their importance in chronic viral infections.

### HBV and HCV Infections

The characteristic dysfunctional immune response in hepatitis B (HBV) and hepatitis C (HCV) infection allows acute inflammation as well as chronification of these diseases (61). Patients have a particularly high risk for liver cirrhosis and hepatocellular carcinoma (HCC), which explains the necessity of optimized treatment options (62, 63).

It is well known that Kupffer cells (KC) as macrophages of the liver induce immune responses to eliminate the virus in acute hepatitis. Though, they hold a dual role in HBV and HCV infection inasmuch as they further initiate immune toleration to limit excessive inflammation, ultimately facilitating hepatitis chronification (114, 115). Unlike acute hepatitis, such dysfunction of CD14^+^ monocytes and macrophages in chronic viral infections is due to increased levels of checkpoint molecules (61, 64). Moreover, HBV^+^ and HCV^+^ macrophages express the M2 immune profile associated with significant decrease of pro-inflammatory cytokines (IFN-γ, TNFα, and IL-12) (64, 65). This macrophage phenotype results in impairment of the host immune response and promotes viral persistence, whereas M1 polarization correlates with pathogen clearance and higher survival rates.

Regarding specific checkpoint molecules in the pathophysiology of these infections, PD-1 and Tim-3 are already shown to inhibit production of inflammatory IL-12 on monocytes and macrophages (64), which explains why HCV-infected macrophages shift towards M2 after checkpoint expression (66). Consequently, increased levels of anti-inflammatory cytokines such as IL-10 further contribute to the “pro-disease” M2 immune profile, ultimately facilitating viral persistence (67). Furthermore, administration of anti-PD-1 antibodies augments IL-12 production by macrophages in HCV-infected patients, likely inducing a shift towards the inflammatory M1 immune profile (68). An even higher benefit is seen after combination of standard antiviral therapy and PD-L1 blockade as this approach significantly enhances IL-12 production, resulting in improved anti-viral immunity.

Similar results are shown for Tim-3. Regularly, its expression by macrophages and DCs is already on a high level. However, in acute HBV and HCV infection, overexpression is detected and correlates with diminished immune cell function in macrophages (64). Overexpression is further reported in chronic hepatitis B and acute-on-chronic liver failure (ACLF) (69). Such upregulated expression levels of Tim-3 promote M2 macrophage polarization, ultimately limiting immune reaction and therefore allowing disease progression (116). Consequently, Tim-3 inhibition rescues compromised immune function and enhances viral clearance (70, 116). Besides that, upregulation of both PD-L1 and galectin-9, ligands of PD-1 and Tim-3, has been reviewed on circulating CD14^+^ monocytes and KC in chronic HBV, liver cirrhosis, and HCC (2). It seems highly possible that co-expression of PD-1, Tim-3, and their ligands contributes to macrophage dysfunction and disease progression as well as chronification (71, 72).

Inhibition of these immune checkpoints represents a promising strategy in the management of HBV/HCV infection, both in single as well as combined application. In addition to current anti-viral therapy, checkpoint inhibition might ameliorate patients’ wellbeing and overall survival rates, even in the chronic state.

### HIV Infection

Human immunodeficiency virus (HIV) infection, another chronic viral disease, is not life-threatening in itself, rather HIV-provoked immunosuppression is the main concerning aspect, because it facilitates subsequent infections that can be fatal. Most of the infected immune cells are eliminated except for macrophages in which HIV survives and that are consequently used as virus reservoirs (73). Hence, there is great need for innovative treatment options focusing on macrophages as virus targets to prevent long-term infection with high viral reproduction (74, 75).

Closer examination of immune checkpoints on HIV^+^ macrophages highlights that PD-1 and its ligands are of particular importance. In rhesus macaques suffering from simian immunodeficiency virus (SIV), the quantity of PD-1^+^ alveolar macrophages (AM) is shown to directly correlate with virus severity, as TNFα expression levels and antibody-dependent phagocytosis (ADP) are drastically reduced in PD-1^+^ AM compared to a PD-1-deficient population. Therefore, it can be assumed that PD-1 expression contributes to macrophage shift towards the anti-inflammatory M2 immune phenotype, allowing HIV to persist (74).

In reaction to HIV virion exposure, PD-1 ligands, especially PD-L1, are increased on macrophages. However, the ligands probably serve different functions as PD-1-mediated expression of anti-inflammatory IL-10 further enhances PD-L1 expression levels, whereas PD-L2 is upregulated following IL-10 blockade (76). It therefore seems possible that dysfunction of macrophages in HIV/SIV infection is because of increased PD-1 and PD-L1 expression, and less likely caused by PD-L2 (74), similar to what is seen in SIV-infected T cells (117). PD-1 blockade on immune cells results in stronger anti-viral immune responses to SIV due to intensified phagocytosis by macrophages (74). Checkpoint inhibition of PD-L1 also causes temporary viral control throughout administration (118). These results imply that PD-1 contributes to substantial alteration of macrophage immune phenotype and morphology, although it cannot explain the complex modulation of HIV infection in all aspects. Certainly, PD-1 ligands are also well involved, but to a lesser extent than PD-1.

Regarding co-regulatory receptors other than PD-1 in HIV^+^ macrophages, there is evidence for Tim-3 inhibiting the release of HIV-1 protein. Besides that, Tim-3 expression results in an inflammatory immune reaction, eventually decreasing viral load; whereas, checkpoint blockade of Tim-3 reinforces the production of HIV (77). Bharaj et al. further reviewed immune modulation by VISTA in HIV infection. Strong upregulation of VISTA on HIV^+^ monocytes causes enhanced secretion of anti-inflammatory cytokines, leading to limited anti-viral immune reaction (78).

Consequently, it is possible to say that disease progression, at least in part, depends on the expression of co-regulatory receptors on infected macrophages. Possible therapeutic interventions for HIV infection might be inhibition of PD-1 and VISTA, stimulation of Tim-3 expression, as well as combinations of the aforementioned factors.

### Influenza A Type H1N1

Besides these significant effects of immune checkpoints in chronic infection, their importance in acute viral infection should also be emphasized. Influenza A infection, an acute respiratory disease, is characterized by inflammation of the upper respiratory tract to varying clinical severity and causes seasonal endemic infections. Regular immune response after viral infection involves upregulation of primarily macrophage-controlled pro-inflammatory cytokines and thereby allows viral clearance.

During Influenza virus infection, enhanced secretion of pro-inflammatory cytokines such as IFN-γ on macrophages is observed, which further increases Tim-3 expression (79). Afterwards however, Tim-3 contributes to a change in macrophage phenotype towards the anti-inflammatory, “pro-viral” M2 type. The resulting minimal macrophage immunity allows persistence of influenza infection (80, 81). By contrast, suppressing the inhibitory function of Tim-3 *via* L3G (a monoclonal antibody against Tim-3) reinforces the production of anti-inflammatory mediators in macrophages and T cells (79). These inflammatory mediators then again activate macrophages, ultimately limiting infection in immune cells.

Consequently, Tim-3 contributes to better understanding of Influenza infection, emphasizing the therapeutic potential of Tim-3 checkpoint blockade.

### Tuberculosis

Co-regulatory receptors also play an essential role in bacterial infections as explained by means of tuberculosis (TB). Overall, the global TB incidence increases while simultaneously an increasing number of multidrug-resistant *Mycobacterium tuberculosis* (MTB) variants occur, resulting in failure of the standard quadruple therapy. Therefore, the antibiotic therapy management urgently needs to be modified (82, 83).

Tuberculosis is a unique bacterial infection as MTB is often not affected by phagocytosis and actually continues to survive and multiply inside macrophages, causing reactivation several years later. Therefore, it is challenging to assume that M1 macrophage polarization is directly followed by pathogen clearance and disease regression, as is seen in most other diseases. It should also be considered that not all macrophages are MTB-infected at the same time; hence, macrophage killing has both beneficial and detrimental effects on patients’ disease course. Thus, various effects are shown for specific immune checkpoints regulating TB infection.

Following upregulation of IFN-γ at the site of TB lesions, macrophages are polarized towards the inflammatory M1 phenotype owing to downregulation of M2-associated CD16 and CD163. These M1 macrophages show increased expression levels of PD-1 and its ligand PD-L1 and are highly susceptible to CD8^+^ T cell cytotoxicity (119). CD8^+^ T cell-caused macrophage killing further results in bacterial clearance, thereby improving patients’ condition. Such cytotoxicity against macrophages can be enhanced by PD-L1 blockade, but it is only effective on IFN-γ-activated macrophages (119). Therefore, ICI of PD-(L)1 presents a potential therapeutical option. Similar findings are reported in macrophages of PD-1-deficient mice as pathogen clearance and antigen presentation are limited, ultimately resulting in high bacterial load, focal necrosis, and low levels of infiltrating immune cells (84, 120). Consequently, PD-1^+^ mice infected with TB show higher survival rates owing to improved bacterial control (85). Besides that, however, PD-1 expression is shown to correlate with reduced levels of macrophage phagocytosis and limited cytotoxicity, resembling the pro-disease M2 phenotype (74). Furthermore, inhibition of both PD-1 and PD-L1/-L2 significantly augments macrophage phagocytosis in active TB, likely due to a macrophage polarization shift towards the M1 type (86). These controversial results emphasize the need for further investigation, especially regarding the unique role of the checkpoint molecule and its ligands.

Sada-Ovalle et al. identify Tim-3 as another even more promising immune checkpoint in the pathology of TB. Inhibition of Tim-3 increases the secretion of inflammatory cytokines such as IL-6, TNFα, and IFN-γ and also reduces production of anti-inflammatory IL-10. This results in pro-inflammatory M1 state, characterized by enhanced macrophage phagocytic activity and thereby, limited bacterial load. Tim-3-ICI has a greater impact on cytokine levels than blockade of PD-1 (121). Therefore, blockade of the Tim-3 pathway likely presents a favorable option in therapeutic management of TB. Inhibition of the Tim-3 ligand galectin-9 on monocyte-derived macrophages (MDM) leads to similarly reduced bacteremia as Tim-3-ICI, although to a lesser degree. However, blockade of both Tim-3 and PD-1 did not provide a more favorable result than blockade of Tim-3 alone; hence, Tim-3 seems much more relevant for TB development (121).

Considering the unique complexity of MTB survival in macrophages, the substantial need for innovative treatment options becomes evident. Apparently, checkpoint modulation on macrophages seems to be a potent approach for consolidating bactericidal activity and restoring innate immunity.

### Sepsis

Sepsis is a systemic inflammatory pathology with high risk of subsequent organ damage and has multiple trigger factors, eventually causing excessive immune reactions. After release of pro-inflammatory cytokines, cascade-like activation of further inflammatory signaling pathways is initiated. Overall, this leads to immune dysregulation because of imbalanced anti-infective and anti-inflammatory reactions.

Focusing on macrophages in septic mice, they mostly present an anti-inflammatory phenotype (87, 122). Such M2-polarized macrophages produce high levels of immune-suppressive, anti-inflammatory mediators such as IL-10, as a reaction to PD-1 and PD-L1 expression. This correlates with the diminished ability to clear microbial invasion in septic mice, because macrophage phagocytosis is impaired (122). Thus, stimulation and overexpression of PD-1/PD-L1 in macrophages of septic patients limits pathogen clearance and contributes to disease exacerbation (88, 89).

Macrophages of PD-1-deficient septic mice, however, have better functional ability than septic wildtype counterparts. Thus, bacterial clearance is enhanced in PD-1-deficient mice owing to adequate phagocytosis and restricted production of inflammatory cytokines (122). Similar findings have been reported in a murine polymicrobial sepsis model induced by cecal-ligation and puncture (CLP). LD01 as a peptide-based PD-1 checkpoint inhibitor significantly improves survival by reducing bacterial burden. This is attributed to higher macrophage phagocytic activity. PD-1 pathway administration can be used to alter bacterial clearance, supposedly because of the change in macrophage polarity. By contrast, PD-1 (over-)expression causes sepsis progression as a result of restricted immune activation that is characteristic of the M2 macrophage profile (90).

Vu et al. explained macrophage polarity following CLP-induced sepsis in considerable detail. On day 1 post-CLP, the levels of PD-1^+^ macrophages are significantly increased, while presenting an exhausted immune phenotype similar to the M2 profile. Besides that, activated macrophages in an M1-like phenotype can only be found on day 12 post-CLP when PD-1 expression is low (91). This confirms a correlation between PD-1 overexpression and M2 macrophage phenotype in sepsis as previously suspected. It is further reviewed that macrophages from PD-1-deficient mice are resistant to sepsis-induced cellular dysfunction, characterized by diminished bacterial clearance, limited inflammatory cytokine secretion, and decreased antigen presentation (85). Therefore, PD-1-deficient murine macrophages have improved survival rates given the M1-like anti-inflammatory immune reaction (122).

Recently, equal results are presented for Kupffer cells (KCs), which are the macrophages of the liver (92). Toll-like receptor (TLR) signaling generally leads to enhanced expression levels of PD-1 in monocytes and macrophages (93). PD-1-expressing KCs show an M2 immune profile known to be rather immune-suppressive, facilitating sepsis progression, as endogenous immune responses are downregulated. However, PD-1-deficient mice showed significantly reduced tissue bacterial burden, lower sepsis scores, and overall reduced disease severity, which can be attributed to M1-like KC phagocytic activity. Similar findings were reported after anti-PD-1 checkpoint therapy in mice with liver injury, strongly correlating with improved KC bacterial elimination and further providing protection from sepsis progression.

These results indicate that PD-1 and its ligands have a massive impact on macrophage function and consequently also on immune reaction to sepsis. Accordingly, checkpoint inhibition presents a promising therapeutic intervention.

## Co-Regulatory Receptors in Inflammation and Autoimmunity

### Multiple Sclerosis and Autoimmune Encephalomyelitis

It has recently become increasingly apparent that apart from cancer and infectious diseases, immune checkpoint molecules are also very significant in inflammatory autoimmune conditions.

Chronic central nervous system (CNS) inflammation results in a variety of neurological symptoms that can until now, only be treated symptomatically. Only minor levels of peripheral immune cells are found in healthy brain parenchyma. In multiple sclerosis (MS) lesions, microglia make up around half of the immune cells and are considered functionally analogous to macrophages. Microglial cells promote MS progression as they are essential for myelin phagocytosis, release of pro-inflammatory mediators as well as antigen presentation (94). Therefore, they display a promising therapeutic target in inflammatory brain diseases (94, 95).

Taking a closer look at specific immune checkpoints in the regulation of inflammatory CNS conditions, microglia are shown to highly express VISTA under physiological conditions. Although VISTA’s function in CNS immune cells are currently not known, its expression is known to facilitate phagocytosis (96) and leads to increased cytokine production (78) and chemotaxis (97) in microglia. While this effect seems to be similar to M1 polarization, different from other diseases, categorizing MS microglia as M1 or M2 is still debatable and has not been useful to date because of functional discrepancies in managing local inflammation (94). Nevertheless, dysregulated VISTA expression affects microglial immune function by inhibiting efferocytosis (96), but overexpression of VISTA on human macrophages enhances bactericidal immunity by stimulating production of inflammatory cytokines (78).

In an MS mouse model of autoimmune encephalomyelitis (EAE) as well as in human chronically active MS lesions, VISTA expression in macrophages is impaired (98, 99). Likewise, inhibition of VISTA causes deteriorated autoimmunity in EAE (100, 123) *via* upregulation of phagocytosis and diminished cytokine secretion (99). These data demonstrate that reduced levels of VISTA contribute to MS disease progression by affecting immune responses, especially microglial phagocytosis (124). Depending on the type of MS lesions as well as their microenvironment, microglia show different reactions and need to be further categorized.

Moreover, the PD-1 pathway generally modulates synaptic transmission, plasticity, and general neuronal function (101, 102). In MS specifically, PD-1/PD-L1 signaling changes microglial phenotype towards an anti-inflammatory type and thereby facilitates anti-disease polarization, similar to the M2 phenotype (103). Induction of such an M2-like immune profile limits production of pro-inflammatory cytokines such as IL-12, which further suppresses immune reaction and hinders MS progression (68). However, in PD-L1-deficient mice a compensatory increase of PD-1 and PD-L2 expression is detected (104), suggesting further regulatory mechanisms regarding the PD-1 axis and its ligands.

Opposing effects are shown for CD47, another recently discovered immune checkpoint of importance in MS pathology (105). By interaction with its ligand SIRPα, CD47 potently inhibits microglial phagocytosis (as previously seen on macrophages) in a variety of other diseases. In MS specifically, CD47 expression is upregulated; hence, decreased phagocytosis allows disease progression.

In summary, CD47 immune regulation in MS and EAE is entirely different from other immune checkpoints, which tend to be upregulated during inflammatory CNS processes. Contrary to the downregulated expression of VISTA and PD-1, that would need stimulation to improve disease condition, limited or no expression of CD47 seems to be a feature of anti-inflammatory state and healthy brain condition; therefore, CD47-ICI offers a promising therapeutic approach.

### Atherosclerosis

Atherosclerosis is a multifactorial disease characterized by vascular damage in all three layers of the arterial walls and lipid accumulation containing malfunctioning macrophages. These so-called foam cells express a wide range of pro-atherogenic cytokines promoting destabilization and growth of atherosclerotic lesions, eventually causing lesion rupture and subsequent cardiac diseases (125, 126).

In general, macrophages present an immense functional heterogeneity of affecting local inflammation in atherosclerosis. Because phagocytosis plays a central role in the pathogenesis, and change of macrophage phenotype is reversible, the simplistic approach of classifying macrophages as M1 or M2 polarized is very inaccurate (127). Instead, alternative categorizations are needed to consider all diverse aspects of macrophage phenotypes.

Examination of specific immune checkpoints in atherosclerosis has shown that CD47 is critically important in the regulation of macrophage immune reactions. Reacting to TNFα- and NFκB-mediated cell activation, unengulfed macrophages in an inflammatory state express CD47 (128). After interaction with ligand SIRPα, effective phagocytosis is constrained and allows disease progression, representing a change towards a pro-atherogenic phenotype (126). It is still unclear whether necroptotic macrophages in atherosclerosis also express CD47, which restricts phagocytosis and thereby promotes disease progression (126). Moreover, expression of CD47 and SIRPα is shown to be significantly higher in apolipoprotein E deficient (ApoE^-/-^) mice than wildtype mice, and positively correlates with the expression of M2-associated markers (e.g., Mrc1) (129). Therefore, it seems that the CD47 expression contributes to the characteristic anti-inflammatory immune profile favoring dyslipidemia and atherosclerosis in ApoE deficiency.

By contrast, limited interaction with SIRPα on macrophages in the aorta of such ApoE^-/-^ mice results in higher levels of phagocytosis (129). Anti-CD47 treatment in a mouse model of atherosclerosis also leads to significantly improved efferocytosis, increased clearance of apoptotic cells, and further reduced size of atherosclerotic lesions (128). Therefore, blockade of the CD47-SIRPα axis stands out as a novel therapeutic opportunity to enhance microglial efferocytosis and prevent disease progression.

Focusing on the PD-1 axis, both PD-1 and PD-L1 are less expressed on myeloid DCs and T cells in patients suffering from coronary artery disease (130). These reduced levels correlate with enhanced pro-inflammatory cytokine production. It is also known that in hypercholesteremic, LDL receptor-deficient (LDLr^-/-^) mice, PD-L1 and PD-L2 are upregulated on macrophages in aortic lesions (131); whereas, PD-1 is overexpressed on aortic T cells (132). Vascular lesions in LDLr^-/-^ PD1^-/-^ mice contain many immune cells that produce particularly high levels of TNFα, contributing to pro-atherogenic immunity. Further, significantly intensified atherosclerosis condition is observed in LDLr^-/-^ mice after PD-1 blockade therapy (132, 133). These findings substantiate that PD-1 regulation affects the phenotypic and functional plasticity of macrophages in atherosclerosis. Nevertheless, further research is needed, with particular focus on the influence of PD-1 ligands on immune regulation.

Likewise, substantial impact is shown for Tim checkpoint pathways. On hypercholesteremic LDLr^-/-^ mice displaying the characteristic pro-atherogenic phenotype, checkpoint blockade of Tim-3 promotes early as well as advanced atherosclerosis (133, 134). Further examination showed that mice deficient in Tim-4 present dysfunctional macrophages that are unable to perform phagocytosis of apoptotic bodies (135). Similarly, mice treated with an anti-Tim-4 antibody have limited efferocytosis performance, contributing to enlargement and infection of atherosclerotic lesions. It can therefore be concluded that high level of Tim protein expression ultimately leads to limited phagocytosis and lower disease severity.

Glucocorticoid-induced tumor necrosis factor receptor family-related protein (GITR) is mainly expressed in human atherosclerotic lesions containing foam cells. Following GITR/GITR-L interaction, human and mouse macrophages produce inflammatory cytokines such as TNFα (136, 137), increasing macrophage function and thereby facilitating disease progression in atherosclerosis. In contrast to the correlation of GITR with disease severity, GITR deficiency on macrophages reduces inflammation and therefore improves condition of atherosclerotic mice (138). This effect is even more significant on ApoE^-/-^ mice, in which deficiency of GITR not only impairs expression of inflammatory cytokines but also reduces reactive oxygen species and minimizes monocyte recruitment to the endothelium (139). Thus, GITR checkpoint inhibition offers substantial improvement for patients suffering from severe atherosclerosis; hence, this approach stands out as a promising immunotherapeutic strategy (140).

Although it is challenging to declare specific treatment approaches given that inflammation and phagocytosis are part of disease pathology, stimulation of PD-1/PD-L1 and Tim-3/-4 as well as checkpoint inhibition of CD47/SIRPα and GITR/GITR-L seem to be promising therapeutic options, preventing subsequent cardiovascular pathologies.

### Type 1 Diabetes

Type 1 diabetes is defined by autoimmune destruction of the insulin-producing beta cells of the pancreas, resulting in micro- and macroangiopathies that cause severe organ damage. Because of secretion of inflammatory cytokines, M1 polarized macrophages phagocytose pancreatic islet cells and thus contribute to disease progression (141, 142).

In a Streptozocin (STZ) mouse model, reduced levels of CD47 were found in the islet cells of STZ-treated mice, consequently followed by reduced interaction with ligand SIRPα (143). CD47 as a “don´t eat me” signal inhibits macrophage-mediated phagocytosis of endogenous cells (144, 145); hence, reduced levels of CD47 and SIRPα result in enhanced macrophage activity and thereby increased disease burden, compared to healthy mice. Furthermore, CD47 inhibition of macrophages also allows progression of autoimmune diabetes, whereas augmenting expression levels presumably improves survival, as CD47 represents a protective agent of pancreatic islet beta cells in inflammation (143, 146).

PD-1-mediated signaling in macrophages similarly contributes to *in situ* immune regulation of the pancreas. Thus, impaired PD-1-PD-L1 interaction and checkpoint inhibition are major trigger factors for disease exacerbation, especially in individuals suffering from β-cell autoimmunity (147).

At the moment, it seems that combined stimulation of the CD47 and PD-1 pathways with agonist antibodies might be a powerful treatment for type-1 diabetes by manipulating substantial features of macrophages so that characteristic autoimmune destruction of pancreatic islet cells is prevented.

## Concluding Remarks and New Directions

Checkpoint modulation is a highly promising strategy to treat cancer pathologies, and its potential is being extensively researched. This allows the development of alternative therapeutical approaches, continuously improving current disease management of various malignant diseases. However, there is still a great need for further examination focusing on different checkpoint molecules and especially their cell-specific influences on underlying pathologies.

It is widely known that expression of certain checkpoint molecules has an influence on immune cells, e.g., T cells. In many diseases, macrophages, in particular, are crucial due to their impact on mobilizing both innate and adaptive immunity, leading to eradication of pathogens. Further convertible M1 and M2 macrophage profiles determine disease development and possible regressions. The focus of checkpoint therapy should thus be extended and include reprogramming of macrophages to enhance immunity in order to destroy disease-causing cells or harmful microorganisms.

Using immune checkpoint molecules as predictive biomarkers on macrophages for malignant, infectious, and autoimmune diseases would allow individual and highly-specific therapeutic options based on diagnostic findings. Such personalized treatments will most likely be superior to the existing broad approaches. However, as immune checkpoints show remarkable functional heterogeneity amongst different diseases and cell types, these diverse pathophysiologic mechanisms have to be considered to achieve successful outcomes.

On the one hand, checkpoint inhibitors are not cell-type specific pharmaceuticals at the moment, resulting in insufficient clinical outcomes for malignant diseases because of inadequate anti-tumor immunity. Often times, even if the cancer burden is reduced, ICI is associated with intolerable suffering due to the severe side effects. On the other hand, only inhibition of immune checkpoints is performed, despite the fact that stimulation of checkpoint expression also seems to be a powerful immunotherapeutic strategy, mostly for use in cases other than malignancies. Even combining inhibition and stimulation of certain disease-relevant checkpoints appears to be beneficial from our perspective.

Nonetheless, paying close attention to the possible reciprocal interaction of checkpoint molecules is essential. As broad inhibition of many checkpoints on all immune cells triggers too many uncontrollable regulations, most of them also currently unidentified, it is necessary to define the exact checkpoints on a specific cell type for therapeutic manipulation. Such specification is inevitable to avoid situations in which these pathways affect each other, and do not allow a precise and foreseeable modulation of corresponding diseases and cause more severe side effects. We are convinced that up- or downregulation of immune checkpoints should ultimately follow cell-specific expression and impact on pathophysiology, considering possible synergisms with further checkpoint molecules. Regarding this context, it seems useful to reflect generally accepted findings of checkpoint molecules on macrophages in e.g., malignant diseases, to comprehend underlying regulatory mechanisms.

In cancer, an insufficient reaction to checkpoint inhibitor therapy can be explained by a low number of tumor-infiltrating T cells, macrophages, or other immune cells (5). However, it can also be caused by upregulation of alternative checkpoint pathways (10). Effective treatment is therefore only possible if the targeted cells express the exact checkpoint molecules that are supposed to be blocked, highlighting the importance of checkpoint expression analysis prior to therapeutic interventions.

Besides resistance to therapy, side effects are of undeniable importance in the administration of immune checkpoint inhibitors. These immune-related adverse events (IRAE) are mostly inflammatory reactions such as hypophysitis, encephalitis, myocarditis, hepatitis, pneumonitis, type 1 diabetes, colitis, myositis, dermatitis, as well as thyroid dysfunction, and commonly occur during combined therapeutical approaches. Owing to synergisms in coregulatory pathways (3), successful reduction of disease burden correlates strongly with worse side effects. Anyhow, adverse inflammatory reactions can also be considered evidence for augmented immune activation against malignancy. Targeting single or multiple immune checkpoints cell-type-specifically on macrophages generally seems to be a more promising way to reduce disease burden and might also limit occurrence and severity of adverse events.

Furthermore, it is important to consider that adverse immune reactions caused by treatment against a disease may be therapeutically beneficial for a different disease and vice versa. As an example, treatment with an inhibitor of checkpoint X on a specific cell type against a defined cancer triggers an autoimmune reaction (e.g., acute hepatitis), implying that for this specific autoimmune condition, stimulation of checkpoint X on the exact cell type may be a favorable treatment option. Then again, for patients at risk for hepatitis, blockade of this checkpoint X should be avoided.

To conclude, treatment of cancer *via* blockade of co-regulatory pathways is already a highly promising approach. Nevertheless, there is more therapeutic potential for this specific immune-regulating group of molecules. Hence, infections affect cancer and vice versa, and regulation *via* immune checkpoints is extremely relevant for both pathologies and may be used for treatment of various acute and chronic infectious diseases as well. Similar immunoregulatory patterns can be adapted for further diseases to establish treatments based on comparable approaches.

Last, there are many diseases in which immune checkpoints seem to be highly important, e.g., parasitic infections such as helminthiasis, toxoplasmosis, and malaria (8, 148, 149), yet further research is necessary to develop effective therapeutic approaches.

## Author Contributions

All authors listed have made a substantial, direct, and intellectual contribution to the work, and approved it for publication.

## Funding

This work was funded by the Deutsche Forschungsgemeinschaft (DFG, German Research Foundation) under Germany’s Excellence Strategy – EXC2151 – 390873048.

## Conflict of Interest

The authors declare that the research was conducted in the absence of any commercial or financial relationships that could be construed as a potential conflict of interest.

## Publisher’s Note

All claims expressed in this article are solely those of the authors and do not necessarily represent those of their affiliated organizations, or those of the publisher, the editors and the reviewers. Any product that may be evaluated in this article, or claim that may be made by its manufacturer, is not guaranteed or endorsed by the publisher.
